# Book Reviews

**Published:** 1830-07

**Authors:** 


### CRITICAL ANALYSES.

Quæ laudanda forent, et quæ culpanda, vicissim

Illa, prius, cretâ; mox hæc, carbone, *notamus.*—Persius.

*On the Diseases and Injuries of Arteries, with the Operations required for their Cure; being the Substance of the Lectures delivered in the Theatre of the Royal College of Surgeons in the Spring of* 1829. By G J. Guthrie, F.R.S., Professor of Anatomy and Surgery to the Royal College of Surgeons, Surgeon to the Westminster Hospital, to the Royal Westminster Ophthalmic Hospital, &c.—8vo. pp. 416. Burgess and Hill, London, 1830.

By his previous works Mr. Guthrie has established his claim to the character of one of the most instructive practical writers of the day upon the subjects to which he has directed his attention. We therefore approach the consideration of the present work with more than ordinary expectation. Mr. Guthrie has shown that he is capable of making the best use of his experience; and we know that few surgeons can have had more extensive opportunities of investigating the diseases and injuries of arteries; for it is only in the dreadful, yet professionally profitable, scenes of warfare, in which he was long so actively employed, that such injuries can be very frequently seen.

Mr. Guthrie's object, in the first part of this work, is to demonstrate the value and importance of that portion of the pathological collection in the museum of the Royal College of Surgeons, which relates to the subject of aneurism, and to prove that the labours and researches of Mr. HUNTER anticipated nearly all the observations which have been made by his contemporaries and successors. In the subsequent part, the nature and treatment of wounds of arteries are more fully considered, and these important points are principally illustrated by observations and cases which occurred during the late war in Portugal, Spain, France, and the Netherlands. It may be proper to add, that the matter contained in this part has been many years announced as preparing for the press, but, although it has not been published, it has been annually promulgated in Mr. Guthrie's surgical lectures for the last fourteen years.

A brief account is first given of the structure and functions of arteries. The force exerted by the heart in propelling the blood into the arteries has been very differently estimated; but it was inferred by J. BELL and others, that the force with which the blood was propelled was so great as to be irresistible, and that no compression could be made on a large artery sufficient to stop the flow of blood through it. This error Mr. Guthrie exposed and refuted in his work on Gunshot Wounds in 1815.

"The power of the heart, whatever it may be able to raise in weight, is so trifling as to the force required to oppose it effectually, as to be very difficult of calculation. If the axillary artery be divided in the human subject, all that is required to prevent hemorrhage, is to place the end of the fore finger accurately over the mouth or orifice of the vessel, and to retain it there with the least possible effort. I have made the experiment at least twenty times on man, and always with success, which induces me to consider the power of the heart as very trifling; being, in fact, only sufficient to propel the blood through open tubes possessing life, and certain indefinable powers which also influence its motion. That these indefinable powers reside principally in the capillaries appears to admit of little doubt; and when these are better understood, a more correct theory of the circulation of the blood may be established than we at present possess. The arteries of the human body, examined during life in every part capable of inspection, show no sign of alternate regular diminution and enlargement. The only observable motion is that of elongation and elevation, which is in fact that of the pulse. When the artery is pressed so as to diminish its canal, the sensation of a fluid passing through it can be felt, and this it is which gave rise to the idea of an alternate contraction and dilatation, which do not exist. An artery appears during life to be larger than it is after death, even when moderately injected. I have conceived that the arteries contain air in an uncombined state, which may assist in keeping them distended, and in facilitating the circulation; but I have not been able to prove it." (P. 8.)

*Of the Alterations of Structure in Arteries.* Arteries are capable, from their texture, of undergoing the various processes dependent on inflammation; but, by a wise provision of nature, without which the life of man would be continually in danger, they are not prone to suffer from it spontaneously. They are, indeed, rarely affected by it in the acute form, and then generally in consequence of injury, or by the extension of disease from continuity of parts.

"An artery will frequently be observed at the bottom of a large wound pulsating regularly, and continuing to exercise its proper functions, whilst all the surrounding parts are in a state of suppurative inflammation. It will sometimes even be seen stretched across a cavity formed by ulceration, surrounded and thickened by a quantity of lymph thrown out on its surface, yet retaining its functions. The inflammatory action has only extended to the cellular texture surrounding it, and to its outer coat, without implicating those within, and the canal only becomes obstructed when these partake of the inflammatory action. The three different structures forming the separable layers or coats of an artery, are not only intended for the due performance of the function of the artery, but constitute, by diversity of texture, a barrier to the progress of inflammation. When this in its acute form has extended to them all, obliteration of the cavity, ulceration, sloughing, gangrene, and mortification are the results, according to the nature and extent of the inflammation." (P. 10.)

The acute attacks of inflammation which affect arteries are of two kinds, phlegmonous and erysipelatous; a distinction Mr. Guthrie first made in regard to veins, and which is of the greatest importance as a pathological fact, death being the invariable consequence of erysipelatous inflammation, whether it take place in arteries or veins. The symptoms of inflammation of the great arteries are very obscure, and the disease itself is one of infrequent occurrence. Mr. Guthrie has seen every artery in the body that could be observed bounding and vibrating in such a manner, in a patient who had femoral aneurism, as actually to raise the bedclothes, when they were loosely applied to any surface immediately over them. He would refer this great and irregular action of the arteries to irritation rather than inflammation of their internal membrane; and it is admitted by the strongest advocates for an increase of pulsation in acute inflammation of the arteries, that in cases in which the preternaturally increased pulsation of the aorta was most distinctly present, no trace of inflammation has been discoverable after death. In children, at the commencement of eruptive diseases, and in hysterical and highly excitable females, we have often observed this excessive pulsation of the arteries, and we have no doubt that it depends upon a peculiar irritability of constitution, and that it is unconnected with inflammatory action. Erysipelatous inflammation of arteries has not been described as an idiopathic disease, but Mr. Guthrie believes that the inflammation which succeeds to an injury, and spreads along the internal coat of an artery until it reaches the heart, is of that nature, and that it is a most fatal disease. He has only verified its existence by dissection in three instances; and in all the patients died very quickly after the accession of the symptoms. In these cases the erysipelatous inflammation of the arteries was evidently caused by continuity of parts with those already inflamed; and in all of them the inflammation was a deep-seated disease, situated in the muscles and internal structure of the thigh, the skin being apparently unaffected.

The experiments of Laennec, Dutrochet, Bouillaud, and Andral, are referred to, to prove that the general tint of redness, which is sometimes seen to pervade the internal coat of all or most of the arteries, is dependent on imbibition, and not on inflammation. "This tinting of the arteries from imbibition is frequently found in persons who have died of putrid fevers, and was even supposed to give rise to a particular kind of fever; but it was also seen in consumptive persons who were long in dying, and in others whose blood did not coagulate. It is not accompanied, or has not been observed to be accompanied, by any particular symptom, from which the presence of such an alteration might be suspected." Upon this point Mr. Guthrie is supported by the opinion of Laennec, who expresses his doubts whether this kind of redness gives rise to general symptoms sufficiently constant or severe to indicate its presence. This eminent pathologist detected this colour of the arteries in subjects dead of very different affections, and he was never able to foretell its existence by any constant signs.* Calcareous matter is frequently deposited on the coats of arteries; and, wherever this may occur,

"It destroys the elasticity of the vessel, and renders the coats so

* Forbes' Translation of Laennec, third edition, p. 657.

brittle, that a ligature will frequently cut through the external as well as the inner ones. The coats of the artery are however so altered by it, and the change which preceded its deposition, that those healthy actions which take place in a sound artery, for its consolidation after a ligature has been applied, do not always occur; and when the ligature is separated, the artery is found pervious, and hemorrhage is the consequence. It was principally from the consideration of this circumstance, that Mr. Hunter was induced to place his ligature in aneurism considerably higher up on the artery than the tumor, with the hope of finding it there in a sounder state." (P. 25.)

It has been said that the internal coats of arteries have been found affected with black spots or tubercles, resembling those which are usually considered melanotic; but Mr. Guthrie has not seen any alteration of this nature. The coats of arteries are subjected to other depositions between them, which have been denominated, from their substance and appearance, atheromatous or steatomatous; but the distinctions between these terms, as applied to diseases of arteries, is by no means well defined or satisfactorily understood. There are few persons past sixty years of age, in whom some change has not taken place in the commencement of the arterial system. It is only, however, when it has proceeded so far as to cause some obstacle to the circulation, that the symptoms of disease present themselves, and are augmented by others, the result of derangement of the neighbouring organs.

"Difficulty of breathing on a moderate effort, such as going up a rising ground or a flight of stairs, first occurs, followed by palpitations in the chest, a severe cough and mucous expectoration, resembling asthma or chronic bronchitis; a gradual failure of strength and appearance, irregularity of pulse, sallowness of countenance, œdema of the limbs, and frequently dropsy or suffocation, lead to the termination of the sufferings of the patient." (P. 34.)

*Of the preternatural dilatation of arteries, and of aneurism.* Sennertus maintained that the internal coats of the artery were ruptured when aneurism occurred spontaneously, or that they were divided, and not reunited, when it took place from injury; and that in both the cellular or external coat was dilated. Scarpa has supported this opinion; but, says Mr. Guthrie, contemporaneous and later observations have proved, from dissection, that neither this nor any of the exclusive theories of the formation of aneurism are correct, there being several ways in which it may occur: a conclusion which surgeons in England might have arrived at thirty years ago, if they had taken the trouble to examine the specimens of aneurism in the Hunterian collection. To illustrate the various species of aneurism, different preparations in the museum of the College of Surgeons are referred to and described.

Mr. Guthrie suggests the following arrangement of the several species of diseases, and their consequences, which he thinks deserves some attention from future observers; aneurism having hitherto been considered to be equally a consequence of all.

"1. That a *preternatural enlargement* may exist with a natural appearance of the coats of the artery, subject, in all probability, to the loss of a part of their elasticity. In some instances, the coats of the artery are thinner than usual, principally at the expense of the middle coat; but, in the generality of cases, the external and middle coats are thicker, and the inner coats softer and more easily detached than is usual from the middle coat. When appearances of more positive disease show themselves, which is not always the case, they are the atheromatous, or the whitish or cartilaginous patch, attended by calcareous deposits in spots and scales; which latter more frequently lead, as the disease advances, to the honeycombed appearance of the inner membrane from ulceration, as in 367 A, page 78.

"2. *True aneurism* is more generally the consequence of the atheromatous or steatomatous disease affecting the middle coat. It may be combined with a cartilaginous state of the artery, and is not free from calcareous deposit; but the two latter appearances are not sufficiently marked to give the same character to the disease as in the preternatural dilatation.

"3. It is difficult to explain in what the difference consists, which exists between the state of the internal coat in *preternatural* dilatation and in *true* aneurism; so that the blood does not coagulate in the former, whilst it is deposited in concentric layers in the latter.

"It is possible that it arises from an excess of refinement in the distinction between the two diseases, and that there ought not to be, and perhaps there is not at a late period, any difference between a preternatural dilatation, bulging to one side of the artery, and a true aneurism, a state which is remarkably well shown in No. 411 H, page 62. At all events, preternatural dilatations of a large size, and departing from the course of the vessel from which they arise, do generally lose their distinguishing character of freedom from concentric layers of coagula, so that the distinction between them, under these circumstances, is lost.

"4. A *false aneurism* is formed through the rupture of the internal and middle coats of an artery, followed by the dilatation of the outer one; or may be merely an advanced stage of a true aneurism, in which the inner and middle coats have been distended or affected at one part, so as to have been removed. This may also take place with the outer coat, in which case the cellular sheath forms the aneurismal sac. In the same manner this may be afterwards removed when it comes in contact with bone; subsequently the bone itself is absorbed, leaving only the superficial fascia, and ultimately the skin as its external boundary, the removal of which gives rise to death by hemorrhage. This kind of aneurism has also been called consecutive, or external mixed false aneurism.

"5. A *rupture* of the whole of the coats of an artery does not depend on the absence of a cellular sheath, or the non-interposition of a sufficient quantity of cellular tissue between the external elastic coat of the artery, and any superficial covering which it may receive from the inner serous membrane of the pericardium, the pleura, or the peritoneum, but, on a particular disease of the artery commencing with the middle and inner coats, which leads to ulceration and rupture, and not to distention. The opening may be either a rent or a hole of various dimensions. In parts where there is a quantity of cellular tissue, it may yet form a *spurious* aneurism. In other parts it gives rise to death by hemorrhage.

"6. An aneurism in young persons is generally the consequence of some accidental injury or disease of the part in which it is situated, and of which the rest of the arterial system does not partake. A preternatural enlargement is more common to elderly persons, and is usually accompanied by a general derangement of the arterial system, and frequently by aneurism." (P. 84.)

In briefly describing the causes of aneurism, Mr. Guthrie remarks, that the predisposing cause has often been attributed to the effects of syphilis, or mercury, or both; but he does not believe that there is any solid foundation for this opinion. "So many persons have all the different diseases to which arteries are liable, who have never had the disease or the antidote, and so many have had the lues venerea and taken large quantities of mercury, without suffering from more disease of the sanguiferous system than their neighbours, that it does not appear to me philosophical to admit such a predisposing cause, merely because the poor who enter hospitals have been found in many instances to have suffered from both."

There are three ways in which an aneurism may proceed to its cure by the unassisted efforts of nature: by coagulation of its contents, by sloughing, or by an accidental pressure of the sac upon the artery. In aneurism, a soft coagulum is formed as soon as the dilatation is distinctly marked, and coagula are soon deposited in concentric layers, which frequently fill up a considerable part of the sac, and in some instances nearly the whole.

"Where the artery is large and the current of blood rapid, as in the aorta, a small coagulum falling into the canal of the vessel could hardly resist its impetus, or, if it did, it would produce nearly instant death. The aorta is therefore never obliterated by this process, but by the slower one of inflammation of its internal coat, effusion of coagulable lymph, and narrowing of the canal; accompanied by a concomitant enlargement of the collateral vessels, so that the circulation may be carried on when the artery is obliterated. This has been known to take place in the aorta, and is the common course of proceeding in every other artery that has sustained an injury, or in which inflammation of an acute kind has been set up. In smaller vessels in the extremities, which are not kept clear by the rapidity of the circulation, two natural processes favoring the cure of aneurism by coagulation have been observed to take place. The first is the enlargement of the collateral branches to such extent as to enable them to maintain the circulation, if the main trunk be suddenly rendered impervious. The second is the effort made to close or shut up the lower openings from the aneurism, or those most distant from the heart: an effort which has been hitherto scarcely considered as a natural one, but which is frequently attempted, and often with success." (P. 89.)

The Hunterian collection supplies several instances of all the openings into an aneurism, save the upper one, having been closed during life; and Mr. Guthrie thinks these specimens establish the fact that, in aneurism of the extremities, nature resorts to this method as a part of the curative process; and that, although she succeeds in effecting it, still more is requisite to complete the cure, by the filling up and obliteration of the tumor. These preparations also show that, when the lower end of the artery has been obliterated, the aneurism has not ceased to increase.

"When the blood in an aneurismal tumor is coagulated in consequence of the impetus and supply from above being nearly or completely cut off by ligature, the ultimate non-obliteration of the sac is so rare an occurrence as never to be expected to take place, although it may be, and often is, delayed for a considerable time. It seems to me equally certain that the cutting off the passage of blood through the lower or further end of the tumor, neither places the sac nor the blood in it under similar circumstances; and that the same processes cannot be expected to occur, unless under particular circumstances which are not at present understood. For instance, it does not appear that the coagulation of the whole of the blood in an aneurismal tumor is the necessary consequence of the closure of the lower opening; and that, as long as this coagulation does not completely take place, and the impetus of the blood remains, the tumor will go on increasing. Its increase may receive a momentary check from the closure of the lower opening, but more is required to complete the cure. This subject will be further considered hereafter: at present it will be sufficient to refer to the instances in the Hunterian collection, in which the lower or further extremity of the artery was closed by nature, without producing a beneficial change in the aneurism. Others are to be met with in almost every author who has written on the subject, as well as in many cases which have been published separately; but the occurrence has always been considered as accidental." (P. 92.)

The spontaneous cure of an aneurism by its removal through sloughing, or sphacelation, was known to the earliest authors: Severinus, Lancisi, and Guattani, each give instances of this kind. Mr. Guthrie has seen three cases, and in each the disease was in the groin.

"The best case which I know of recovery by sloughing was in the instance of a soldier, under the care of Mr. Albert, in the York Hospital. The tumor was of a large size, and extended above as well as below Poupart's ligament. It pulsated violently; the walls were thin, the surface inflamed. At last the integuments on the apex of the swelling became of a livid colour, pulsation ceased, and the whole tumor became black and flaccid. A small opening formed in the centre, from which fetid coagulated blood issued. The mortification extended around the circumference of the tumor in every direction, and beyond it, until the whole sloughed out. Several pounds of coagulum were discharged, after which the surrounding parts began to take on a healthy action, granulations sprang up, and the part ultimately healed. (P. 97.)

In the other two cases the patients died; not, however, during the sloughing process, but subsequently, being worn out by the continuance of the discharge, and the irritation arising from the ulceration having extended to the hip-joint.

"The artery was obliterated above and below the ulcerated part, and was deficient in the situation of it. It is fair to suppose that both these patients would have recovered, if ulceration had not implicated the ligamentous structure of the hip-joint. Mortification is, however, so dangerous and uncertain a process, so prone to recommence when it has become stationary, and to spread again with the greatest rapidity, that the attempt at a cure by sphacelation must always be viewed with the greatest anxiety, and, if possible, be anticipated by the necessary operation, when situated in parts admitting of its performance. The operation should never be had recourse to after mortification has begun. If the patient dies of the mortification, the operation is useless; and, if he survives it, the operation is unnecessary; for the artery must and will be obliterated for some distance above the aneurism, by the process of inflammation, which necessarily takes place in the separation of the dead parts from the living; and it may, by inflicting an additional injury, prove destructive." (P. 97.)

Accidental pressure of the sac upon the artery, producing the cure of aneurism, has never been seen by Mr. Guthrie; neither has it, he says, been proved by dissection to have taken place. He considers it, therefore, as a theoretical opinion as to the manner in which a cure of aneurism may be effected. Mr. Hodgson, however, mentions some facts tending to show that aneurism has been thus spontaneously cured.* There are several preparations in the Hunterian collection which show that different arteries must evidently have suffered considerable compression, under which *they have altered their direction;* but there is only one in which the artery is obliterated: it is No. 381, an aneurism of the left carotid.

In the next sections, the symptoms of internal, and the symptoms and diagnosis of external aneurisms are detailed. Mr. Guthrie's remarks upon these important points are highly interesting, and we regret that our limits confine us to the following extract:

"There are few surgeons in London who have not seen one case at least of bronchocele, which has been mistaken for carotid aneurism, from inattention to the motions of the tumor, as dependent on those of the larynx. A gentleman, an adjutant of militia, came some few years ago to me, with an enlarged lobe of the thyroid gland, to be operated upon for carotid aneurism: it was cured by iodine. Twice have enlarged and suppurating absorbent glands, nearly in a similar situation, been brought to me for aneurism; and twice have I removed a tumor adhering to the radial artery, which was considered to be of a similar nature. The same thing happened in the groin; and it is almost unnecessary to say that, from inattention, both herniæ and aneurisms have been opened for common suppurating abscesses. I saw this mistake once made in hernia with the best effect. The surgeon punctured the tumor, which was incarcerated, hard, and painful, with a lancet, (fortunately the opening was but a puncture,) and was very much alarmed to find a watery, fetid fluid evacuated, followed by a little fecal matter. He closed the opening by a piece of adhesive plaster, placed a compress upon it, and the patient recovered without any further bad symptoms.

"Pelletan, among other authors, gives two instances in which aneurisms of the axillary or subclavian artery were opened, and the patients died of hemorrhage in consequence. In one case the parts were carefully dissected, and the axillary artery immediately below its dilatation was in so impervious a state as not to admit the smallest probe, and looked like a ligamentous cord. At the bend of the arm it resembled an artery in appearance, but its canal was so contracted that nothing could be introduced into it. If the

* On Diseases of Arteries and Veins, p. 107.

engraving given by Pelletan represents the disease correctly, it was a case of preternatural enlargement, with a consecutive false aneurism. Scarpa, and most other authors, notice similar unfortunate accidents." (P. 114.)

*On the medical Treatment of Aneurism.* The medical treatment of aneurism is now almost entirely confined to those which are internal; or to external ones in which an aneurismal diathesis is presumed to prevail, rendering the cure of one tumor by operation useless, when others of more importance are known to exist, beyond the reach of art. It is only when external aneurisms are complicated with disease of the organs within the chest or abdomen, or are attended by anomalous symptoms of derangement in those parts, or are numerous, that this diathesis can be fairly said to exist; and that internal or palliative treatment is to be preferred to the external or curative process by operation. The internal treatment has not undergone any improvement since the time of its inventors, Valsalva and Albertini. The practice has not maintained its ground as a curative process, but has fallen into disuse from its severity.

" It consists, first, in placing the patient in a recumbent position, and keeping him on as small a quantity of the least nutritive food and drink as is consistent with the support of life. Secondly, in abstracting blood from day to day, first in large quantities, and afterwards in smaller ones, until the force of the circulation, and the circulating medium itself, are reduced to the lowest possible degree consistent with the safety of the patient. Thirdly, the administration of such remedies as will assist in reducing the action of the heart and arteries; whilst they act at the same time on their intimate structures, inducing in them a more rigid contraction and a healthier action : digitalis, acids, and the preparations of lead, are those most relied upon for these purposes. Fourthly, the application of cold externally, by means of ice, snow, or evaporating liquids or preparations ; and the use of bandages, where their application is admissible." (P. 122.)

In the prosecution of this plan, the constitution of each person must of course be studied, and his powers of resistance well calculated, so that they may not be reduced below that level from which they cannot recover. If no improvement take place in the aneurism, the treatment is not to be persisted in.

" Digitalis is a remedy which may be given with advantage in the first instance, but it is one which not only lowers the action of the heart in a remarkable manner, but which will also sometimes put a stop to it altogether. I have known it do this when given in large and repeated doses for the cure of tetanus; and I

am therefore not disposed to recommend its use after the action and power of the heart have been considerably diminished." (P. 124.)

The mineral acids and the superacetate of lead have probably been recommended because they have been known to do good in acute and chronic hemorrhages. Mr. Guthrie has not seen the mineral acids exert any very powerful influence in these cases; but he has often found the superacetate of lead a most valuable remedy in both, when combined with opium. In the proportion of a grain to a quarter and to half a grain of opium, three or four times a day, he has found it very useful, and he has given it in much larger doses without any bad effect. The American practitioners employ this medicine in larger and more frequently repeated doses than are common in this country. Dr. Dewees,* for example, recommends it in uterine hemorrhage, in two or three grain doses, guarded with opium," every half hour or hour, according to circumstances. If the stomach is irritable, he advises it to be given per anum: "twenty or thirty grains may be dissolved in a gill of water, to which will be added a drachm of laudanum." To the application of pounded ice in external aneurisms, Mr. Guthrie is not very favorably inclined. He states, however, that instances are recorded by Larrey, Ribes, and others, in which it seems, when steadily persevered in, to have effected a cure. It is said, also, that, under a continued use of it, mortification of the part has supervened.

*On the collateral circulation.* By this term surgeons understand, in cases of aneurism or wounded arteries, the means whereby blood is sent to the extreme parts of the body or to a member, when the supply through the principal trunk or vessel is cut off, either by disease, injury, or ligature. In the present day it is believed that the collateral circulation is sufficient in every case to support the life of the extremity; and, when it does not do so, the failure is attributed to some accidental defect, rather than from any regular incapability on the part of nature. This fact was long doubted; but Mr. Guthrie satisfactorily shows that surgeons of former times placed no confidence in the power of the collateral branches to support the limb when the main trunk was cut off, because their mode of operating, and their subsequent treatment of the wound,

* Dewees' Midwifery, p. 412. Upon this subject we may also refer to the paper of Mr. Gardner on Lead, at page 20 of this Number.

were not such as is now adopted, and did not afford the patient a fair chance of recovery.

"The great success which has attended the practice of the present day in the operations for aneurism, has, however, led to an overweening confidence in the powers of the collateral circulation, which it assuredly does not under all circumstances possess. The older surgeons did not perceive, in their full extent, the disadvantages under which their operative processes laboured: modern ones have overlooked, in the consideration of this subject, the various advantages they, on the contrary, enjoy in the cases of aneurism on which they have operated. They adopted hastily, in consequence, the theory of the operation for aneurism in the treatment of wounded arteries, not only with relation to collateral circulation, but to other more important points in which it was not admissible. The result of this practice has been a want of success nearly as marked in cases of wounded arteries as it was the reverse in aneurism. The causes of this failure also require explanation." (P. 130.)

The collateral circulation is more perfect, more active, in young persons, during the increase or growth of the body, than it is either at maturity or in the decline of life. Every action is carried on, in consequence, with more facility and certainty, and a greater dependence can be placed both on the powers of the part and of the constitution, than at any other period. Observation has proved the correctness of this statement; and in cases of aneurism, or of wounded arteries, in which the principal or only trunk has been cut off, the collateral branches have been found to be more efficient between the ages of puberty and of the first period of manhood, than at any subsequent time of life. In old age there are many reasons why the powers of the smaller arteries should be comparatively defective, which Mr. Guthrie does not deem it necessary to dwell upon, as the fact is admitted. The important point is not, however, alone referable to the time of life in which the operation is performed, but to the nature of the disease or injury, and the period of time after the reception of one or the commencement of the other that the operation is done, and the call is made on the collateral circulation to maintain the life of the part. Dissection after the successful performance of the operation for aneurism proves two points: that arteries known to exist have become very much enlarged, when compared with those of the opposite side; and that others, not discoverable in the sound limb, have been developed in a similar manner, and both together to such an extent as will nearly, if not quite, compensate, by a circuitous route, for the loss of the direct supply. The principal object of inquiry is, do these vessels always exist? or at what period of time do they begin to enlarge, so as to enable them to carry on the circulation in the manner in which it is presumed to be done?

"The fact of the development of the vessels having taken place after the commencement of the disease, or the reception of the injury, being established, the question of time is now to be considered. Does it commence with the disease, take place during its progress, or only after the operation? I am of opinion that the collateral branches begin to enlarge shortly after the commencement of the disease, as a part of the curative process which nature endeavours to set up in most instances; the essential points of which are, in an extremity, 1st, the obliteration of the artery above and below the tumor; 2d, the coagulation of the blood within it; 3d, the enlargement of the collateral branches above and below it.

"When a limb is lost through mortification, as a consequence of a division or obstruction of the principal artery, it usually takes place after the infliction of a sudden injury, in consequence of the collateral branches not having had time to enlarge." (P. 138.)

Mr. Guthrie adduces various facts which show that the collateral circulation is not in the same stage of preparation, in a limb suffering from a divided artery, as in one in which an aneurism has for some time existed; and they also show why mortification is more common after wounded arteries than after operations for aneurism. From these facts arise three practical deductions:

"1. That the theory of the operation of aneurism, as dependent on the collateral circulation, cannot be applied with safety to aneurisms dependent on wounded arteries.

"2. That it is inapplicable to wounded arteries.

"3. That the length of time a spontaneous aneurism has existed is of consequence, as connected with the collateral circulation; although an aneurism should never be allowed to attain that size which may render it injurious to the surrounding parts.

"I shall remark, in conclusion,

"1. That the collateral vessels are at all times, and under all natural circumstances, capable of carrying on the circulation in the upper extremity, whatever disease or injury may affect the principal trunk. Whenever the reverse takes place, it is an exception to the general rule.

"2. That, after operations for aneurism in the lower extremity, the collateral branches are almost always equal to carry on the circulation through the limb.

"3. That, when the principal artery of the lower extremity is suddenly divided, without any previous disease having existed, mortification is not an uncommon occurrence, and is more likely to take place in old than in young persons.

"4. That when, under such circumstances, the principal vein is also divided, mortification seldom fails to be the consequence." (P. 140.)

*On the surgical treatment of aneurism.* Guattani first recommended general compression of the limb in which an aneurism was seated, but the practice has been abandoned from its want of success. Direct or immediate compression on the artery is effected in two ways: 1, by pressure made on the artery through the integuments, by means of a compress, or by a pad affixed to a spring, which confines the pressure to the spot immediately over the artery, without impeding the collateral circulation; 2, by laying bare the artery, and compressing it with a spring or forceps, or by the application of a temporary ligature, to be removed at the expiration of a certain number of hours.

"The application of pressure by means of a spring pad has been often tried, and has sometimes, although very rarely, succeeded. The process is long, the pain great, and there is danger of the part sloughing. The pain is, however, so great, that few persons can be persuaded to submit to it; and those surgeons who have tried it once will not again put it in competition with the operation. Mr. White, one of my colleagues at the Westminster Hospital, and who is an excellent surgeon and an able operator, tried it in a case of popliteal aneurism in a woman, by means of a newly-invented spring, supposed to possess peculiar advantages. The woman bore the pain heroically for five days, but the parts compressed sloughed deeply. The cure was completed; but the pain, danger, and risk incurred were infinitely greater than any which could have been sustained from the usual operation. I watched the progress of the case with great attention, and will not be easily induced to use that or any other instrument for such a purpose.

"The application of a solid instrument to the artery, for the purpose of compressing it for a certain time until inflammation shall have taken place; or that of a temporary ligature, to cut through the inner coats of the vessel, and then to excite inflammation; have been tried in different ways by a variety of surgeons. The forceps, pincers, or presse-arteres, are the oldest inventions, and the worst of the two; but both have been found inferior to the single small round permanent ligature, and have been consequently abandoned." (P. 142.)

Mr. Guthrie clearly shows the futility of the attempts which have been made to wrest from John Hunter the merit of having first suggested the operation for aneurism, as it is now practised, and the principles upon which it is founded. If this operation and its principle be compared with those performed by Anel and Desault, there will be seen to be little resemblance between them. The operation performed by Anel was forgotten, and it was done not on a diseased, but on a wounded artery, and *close* to the swelling. Desault's was done on a diseased artery, was unfortunate, and was so far considered as an unsuccessful experiment, that it was never repeated by himself, although he lived many years afterwards: on the contrary, he adopted, instead of it, the operation recommended by Hunter. The theory of the operation of Hunter, as performed in the present day, for aneurism, and applied to all the arteries of the body, is

"1. That the ligature be applied as far above the aneurismal sac as the first large branch or branches given off from the trunk of the vessel; and, if necessary from disease, even above them, so as to secure its application, if possible, on a sound part of the artery.

"2. That the aneurismal sac will retain any blood which may be in it at the time of the operation, or any that may come into it afterwards, until that blood has coagulated, and filled up the cavity of the sac, and the orifices of the vessels leading into it; a result which will inevitably take place, unless the collateral branches communicating with the artery above the sac are so large as to be able to pour the blood into it, with an impetus derived directly from the heart.

"3. That the collateral branches, even when some have been cut off above the sac, are yet competent to carry on the circulation in the extremity.

"4. That after the circulation through the limb has been re-established, and the blood been coagulated in the sac, the absorbents will remove it, and reduce the sac to the state of a ligamentous and impervious cord, and restore the limb nearly to its natural state." (P. 151.)

Mr. Hunter did not expect that, by this mode of operating, the supply of blood to the aneurism would be completely cut off: on the contrary, he only says, "The force of the circulation being thus taken off from the aneurismal sac, the progress of the disease would be stopped; and he thought it probable that, if the parts were left to themselves, the sac, with its contents, might be absorbed, and the whole of the tumor removed; which would render any opening into the sac unnecessary." Two cases are quoted by Mr. Guthrie to show the failure of this expectation: the first led to amputation and death; the second, under the care of Mr. Briggs, to a mode of treatment which was successful, "and cannot fail to attract attention, as well as to be imitated in practice."

"James Mack, carpenter, aged thirty years, in February 1829, felt pain in the toes, and ankle, and knee, and afterwards a swelling like a pigeon's egg in the ham, which gave him great pain, particularly at night, and pulsated like his heart. The operation was performed on the 6th of March, by Mr. Briggs. The ligature came away on the 27th, the pulsation in the tumor ceased, and it diminished very much in size. It was only in June he was able to go to his work, when the swelling had entirely disappeared. In September it began to appear again with pulsation, both the swelling and the pulsation being soon greater than before. A compress was applied with a bandage on the part and on the leg, which made it worse. He then applied a hard compress, being in fact a narrow roller, four inches in length, on the inside of the thigh, just above the knee, and above the inner hamstring muscles, which was firmly retained in that situation night and day. This gave him relief by taking away the pain, which was principally felt in the toes and ankle. This compress he wore for two months, at the end of which time the pulsation had ceased, although the swelling had not entirely subsided. He has gradually improved ever since, although he has still pain in the back of the leg, and, on walking, he feels the foot, and particularly the great toe, numb and cold. He cannot quite straighten the limb, and walks with a little halt. There is no appearance of the swelling now, one year after the operation." (P. 157.)

In our succeeding Number, we shall terminate the analysis of this interesting work. We could not have done justice to it in one article. See p. 243.

---

*Observations on the Disorders of Females connected with Uterine Irritation.* By Thomas Addison, M.D. Assistant Physician and Lecturer on the Theory and Practice of Physic at Guy's Hospital.—8vo. pp. 96. Highley, London, 1830.

As these observations were originally delivered as a clinical lecture to the pupils at Guy's Hospital, the author has, of course, confined himself to a brief and practical discussion of the subject he has selected. Upon such an occasion, speculative doctrines would have been out of place. The object of this lecture is to describe the morbid effects produced upon the general constitution, and upon particular parts of the body, by continued uterine irritation. By the term irritation, Dr. A. implies a disturbance in the endowments or functions of a part, without either inflammation or organic lesion being *necessarily* present. In thus characterizing uterine irritation, he does not deny that it may be coexistent with, or even give rise to, inflammation or organic lesion; but, speaking generally, he is inclined to the opinion that it is found to exist altogether independently of either of these states.

"The most frequent symptoms of uterine irritation are *irregular menstruation*, the discharge being preceded or accompanied by pain in the back, loins, or thighs, or in the region of the uterus itself, and attended with forcing or bearing down; the *discharge being in excess* either in point of mere quantity or in continuance, or in recurrence; *tenderness of the womb itself upon pressure* made either externally or *per vaginam*, a tenderness in some instances so great as to interfere with the privileges of matrimony; and, lastly, *leucorrhœa*. The most frequent symptoms, however, are, unquestionably, *painful menstruation* and *leucorrhœal discharge*, although the former is often the only symptom acknowledged by the patient herself." (P. 11.)

The most powerful predisposing cause of uterine irritation is constitutional irritability, especially in persons naturally of a delicate frame of body. Sedentary and luxurious habits, late hours, and passions of the mind, are also enumerated as predisposing causes, as they tend either to produce or to increase morbid susceptibility. The exciting causes are, active exertion of any kind during the flow of the menses; frequent child-bearing, especially if the patient suckle her children herself; excessive venery, and indeed venereal excitement of any kind. From the continuance of uterine irritation, various morbid conditions of the general system and of particular organs result, which vary in kind and degree, according to differences in particular constitutions, and according to the susceptibility of individual organs.

"The first complaint usually made by a female suffering from this irritation, is of feeling *nervous*, probably with a disposition to be low-spirited; but if, in a well-marked case of the kind, you proceed to feel the pulse, and especially if it be your first visit, the chance is that you will perceive a distinct tremor of the hand, and a remarkable acceleration and sharpness of the pulse, evidently arising from mental agitation; a degree of mental agitation so great, in a naturally susceptible female, that if you attempt to soothe or encourage her, she will begin to sob, her lips quiver, and she bursts into a flood of tears. Even after she has sufficiently recovered her self-command, she experiences considerable difficulty in describing her feelings and sensations, and often appears to dispair of satisfactorily communicating to you the nature of her ailment. She tells you that, without any assignable cause, she gradually declined in health and spirits; that she had lost her wonted alacrity, has become indolent, and is easily fatigued by comparatively slight exertion; that she is readily flurried; that her heart often beats, flutters, or palpitates; that the impressions made upon her mind are altogether disproportionate to the causes producing them; that she is very prone to weep, and occasionally experiences sudden and transitory feelings of alarm and dread, especially during the night, without being able satisfactorily to account for them; in short, that both body and mind are in a morbidly sensitive condition, whilst general distress is strikingly depicted in her pale or dejected countenance. If you proceed in your inquiries to ascertain the state of the uterus, you are perhaps informed that she is regular; but if interrogated more narrowly, it will almost uniformly be found that she suffers pain, either before or during the flow, in the back or loins, and that in the intervals she is troubled with leucorrhœal discharge. To these inquiries she gives a reluctant reply; will often, perhaps from delicacy, conceal the truth, or, if she acknowledge it, will probably add, 'Oh, that is of no consequence; that is not my complaint; I have long been accustomed to that, and it does me no harm;' and then winds up the case with telling you that she has taken a load of tonic medicines without benefit. If you ask her whether such questions were ever put to her before, you are generally answered in the negative. Such a case as this, with slight modifications, is of common occurrence, yet it most frequently happens that, with the general disorder, we have decided derangement of some internal organ or organs; and, of these, the organs of digestion appear to be almost uniformly the first to participate: indeed, the derangement of the digestive organs, to a greater or less extent, is so commonly associated with the general affection I have described, that one cannot but conclude that the general affection is most materially influenced, if not in part produced, by it. It is sufficient, however, for our purpose to know that the exalted susceptibility of the general system and this deranged condition of the first passages, are very commonly co-existent, however they may stand in the relation of cause and effect." (P. 14.)

There is frequently a tendency to flatulency, and sometimes the patient suffers from repeated vomiting; paroxysms of hysteria, also, are not unfrequent. In a practical point of view, the most important effects of uterine irritation are pains, varying in degree, in different parts of the body, but especially the abdominal viscera.

To these symptoms Dr. Addison particularly refers, and the subject is one of peculiar interest to the inexperienced practitioner, inasmuch as he is very likely to suspect the existence of local inflammation when it is not present, and consequently to institute what he conceives to be an appropriate treatment, by free bleeding, &c., which will generally inflict positive injury upon his patient, without much chance of relieving her sufferings. We have so frequently touched upon this subject, that we do not think it necessary to dwell more particularly upon the observations made by Dr. Addison: he corroborates the opinions which Dr. Marshall Hall, and various other writers, have advanced upon the same subject. We must observe, however, that, in delicate and highly susceptible females, who are liable to hysterical affections, these wandering neuralgic pains (for such we presume them to be,) are very common, when there is no evidence whatever of uterine irritation.

Dr. Addison observes, that pain affecting the abdomen generally is by no means of rare occurrence, and that in some instances it so closely resembles general peritonitis as to be mistaken for and treated as that complaint. "The pain is complained of over the whole of the belly, and the slightest touch in many instances cannot be borne, such is the extreme sensibility and tenderness of the parts." This is very true; but we would add, that if, from the "slightest touch," firm pressure be gradually made in such cases, the patient no longer complains. The increased sensibility appears, in fact, to be superficial, and hysterical women will often shrink from slight pressure being made on the surface of the body, when they will bear, without complaint, any part to be firmly pressed upon.

Dr. Addison offers some judicious remarks upon the diagnosis, or the means of distinguishing the painful or neuralgic affections he has described from the inflammatory disorders to which they often bear so close a resemblance, and for which they are so repeatedly mistaken.

"Knowing then that such cases are of frequent occurrence, whenever a female complains to you of pain under the left breast, with or without palpitation or pulsation of the heart; of pain in the right hypochondrium; in the situation of the left or right colon; or of acute pain generally over the whole belly, or in the region of the kidneys or bladder; always be upon your guard, and if, on inquiry, you find a few or many of the constitutional symptoms I have described, together with indications of uterine irritation, as shown by pain in the pelvis, in the loins, or in the thighs, before or during the catamenial flow; by too frequent or too profuse menstruation; or by leucorrhœal discharge; I say, when you find such an assemblage of symptoms and circumstances, your suspicions will amount to a high degree of probability that the complaint is not of an inflammatory nature. The comparative absence too of fever, the appearance of the tongue, and the state of the surface, will materially assist you in the diagnosis; although it must be confessed that peritoneal inflammation often exists with but little developement of the general symptoms regarded as characteristic of the phlegmasiæ; whilst, on the other hand, the painful affections I have been describing are not unfrequently attended with an accelerated and vibrating, or even a hard pulse, a hot skin, and furred and slightly brown tongue. Generally speaking, however, the pulse varies much in the latter, and seldom has much hardness in its beat, although, from the irritability of the heart, it is sometimes sharp or jerking, whilst the skin is usually warm, rather than hot, and with a disposition to moisture; and, lastly, the absence of many, or of some of the symptoms of the phlegmasiæ simulated, will go far to complete the marks of distinction." (P. 30.)

That the practitioner, who infers there must be inflammation because there is local pain, will be constantly committing the most egregious blunders, we can readily conceive; but we really think that, in the great majority of these cases to which Dr. Addison refers, but a moderate share of practical experience and judgment will be required to discriminate between these attacks and visceral inflammation. The chief distinction which we would insist upon is the occurrence of hysterical symptoms, as they are called. It has been very truly said by Dr. Marshall, that symptoms of hysteria rarely, if ever, appear while the active inflammation of a vital organ is going on.* The manner in which the patient expresses her suffering is also generally quite different from that which is seen when local inflammation is present. In the latter case, she moves gently and with dread, her countenance at the same time betraying great anxiety and suffering. During these neuralgic or hysterical pains, on the contrary, the patient screams out suddenly and vehemently, rolls about her bed at one moment in the greatest apparent anguish, and at the next becomes perfectly tranquil. In some of these cases, indeed, local inflammations may be so closely simulated as to lead to doubt and perplexity, even with experienced and observant practitioners, but our experience teaches us that such instances are but of very rare occurrence.

*Treatment of uterine irritation, and the disorders connected with it.*

"The indications are, 1st, to correct the morbid condition of the uterus. 2dly. To remove or mitigate the violence of troublesome symptoms in any individual case; and 3dly. To restore tone and vigour to the general constitution." (P. 33.)

In thus laying down the indications of cure, it will be observed that Dr. A. has in some measure reversed the ordinary mode of procedure: he believes that the condition of the uterus, to the continuance of which he ascribes the various disorders he enumerates in his lecture, has generally been overlooked, or at least, if observed, it has only been in those cases which have had some of the characteristic symptoms present, in a strongly-marked degree, such as

* Dr. Marshall Hall on some Diseases of Females, p. 114.

excessive or very painful menstruation, or profuse leucorrhœal discharges; "and even then the condition of the uterus has been usually looked upon as a mere effect or consequence of the nervous or hysterical state of the entire frame; or, in other words, it has been considered as merely participating in an universally morbid sensibility."

That uterine irritation occasionally gives rise to the symptoms mentioned by Dr. A., we readily admit; but we believe that, in general, the uterine disturbance must be regarded, not as the cause, but as the effect, of local or general derangement of health. Dr. Addison, however, takes a different view of the subject, and modifies his practice accordingly. With him the principal indication is to correct the state of the uterus, "although, of course, the second indication, that of allaying troublesome symptoms, must often go hand in hand with, or, in aggravated cases, even take precedence of it." Dr. A. admits that local or general depletion in plethoric subjects, and anodynes and laxatives, in most cases of uterine irritation, may be serviceable and necessary;

> "Yet, speaking from experience, this object, this grand object is secured with much greater certainty, and much more speedily, by applications made directly to the uterus itself, and parts adjacent. The applications to which I allude, are cold astrigent washes, injected per vaginam by means of a proper syringe. The ordinary womb syringe answers the purpose exceedingly well, but one of any convenient shape may be used, provided it be sufficiently large to contain from four to six or eight ounces of fluid. The injection should be introduced with such a degree of force as shall secure its application to the upper part of the vagina, and to the os uteri; and the operation should be repeated two, three, or four times a day, according to the circumstances of the individual case." (P. 36.)

The mineral astringents are preferred, as they do not stain the patient's linen, and are therefore not so much objected to. The wash Dr. A. most frequently employs is the Liquor Aluminis comp. of the London Pharmacopœia. This practice must be persevered in for a length of time, according to the obstinacy of the case, and the effects it produces. Before and during the menstrual period, the use of the injection must be discontinued, and the patient must be cautioned against using violent exertion or undergoing any unusual fatigue at that time, as nothing so completely thwarts the object of the practitioner as imprudence committed whilst the irritable uterus is performing its functions.

"When the uterine irritation is characterized by frequent and excessive flow of the menses, I have directed the patient to remain quiet in or on the bed, and to desist from the wash during each recurrence; not having ventured to carry the practice to the extent of attempting to restrain even such excessive discharge by local astringents. But when there is decidedly painful menstruation, or pain felt in the womb, loins, or thighs before the appearance, although there be no leucorrhœal discharge whatever in the interval, I nevertheless apply the cold wash to such subjects, precisely in the same manner as if such leucorrhœal discharge were present, on the principle that this leucorrhœal discharge is itself a mere symptom or effect of the state of uterus and neighbouring parts, which I am anxious to remove." (P. 38.)

The second indication must, of course, be variously fulfilled, according to the circumstances of each particular case. Bleeding, especially in large quantities, and active and debilitating purgatives, are not advisable for such patients, as they are always in a weak and irritable state. Castor oil, when it agrees with the stomach, will be found more generally serviceable than any other purgative. Sometimes the warm and resinous purgatives may be prescribed with advantage. When the patient is troubled with flatulence, ammonia with magnesia, to which may be added a little tincture of hyoscyamus or hops, will be given with advantage. If, with more or less of the general symptoms, there is pain under the left mamma, or in any of the other situations pointed out, mild sedatives, with or without ammonia, according to circumstances, are recommended; the uterine irritation being attended to at the same time, and the injection before mentioned being employed. Dr. Addison justly laments that this local pain is frequently mistaken, and the patients "blooded, cupped, and blistered, and anointed with acrid ointments, for months in succession, not only without relief, but with the most serious injury to their general health, whilst the uterine irritation has been altogether overlooked."

In pointing out the non-inflammatory nature of this affection, it is not, however, contended that bleeding, cupping, or blistering, is improper in every instance. "On the contrary, in plethoric subjects, one or two general or local bleedings may occasionally prove of service." When the pain shifts its seat from time to time, Dr. A. limits his *external* remedies generally to hot anodyne fomentations. The internal exhibition of opiates will often be required; and if the pain seizes the track of the colon or the stomach, and is accompanied by severe spasm, a full dose of Liq. Opii Sed. or even a full dose of Ether. The practice of giving tonics and stimulants in cases of excessive menstruation, on the erroneous supposition that such discharges are the result of weakness, is deprecated: here vascular excitement is to be allayed by moderate depletion and purging, and irritation to be allayed by anodynes and rest. Menorrhagia sometimes, however, depends upon debility and want of healthy tone in the general system, and then a tonic plan of treatment is demanded.

The third indication is to restore strength and vigor to the general habit. As Dr. A. considers the local uterine irritation to be the source from whence the whole mischief proceeds, he, of course, pays especial attention to the removal of it. The constitutional symptoms are very various, and must be treated upon general principles. In further illustration of his views, Dr. A. relates several instructive cases.

