# Erratum in the Last Number

**Published:** 1830-08

**Authors:** 


ERRATUM *in the last Number.*—In the running title of each page, from p. 65 to 71, for "Mr." read Dr. Addison.

